# Proteomic profile of melanoma cell‐derived small extracellular vesicles in patients’ plasma: a potential correlate of melanoma progression

**DOI:** 10.1002/jev2.12063

**Published:** 2021-02-11

**Authors:** Monika Pietrowska, Aneta Zebrowska, Marta Gawin, Lukasz Marczak, Priyanka Sharma, Sujan Mondal, Justyna Mika, Joanna Polańska, Soldano Ferrone, John M. Kirkwood, Piotr Widlak, Theresa L. Whiteside

**Affiliations:** ^1^ Maria Sklodowska‐Curie National Research Institute of Oncology Gliwice Poland; ^2^ European Center for Bioinformatics and Genomics Institute of Bioorganic Chemistry PAS Poznan Poland; ^3^ UPMC Hillman Cancer Center University of Pittsburgh Cancer Institute Pittsburgh Pennsylvania USA; ^4^ Department of Pathology University of Pittsburgh School of Medicine Pittsburgh Pennsylvania USA; ^5^ Department of Data Science and Engineering, Silesian University of Technology Gliwice Poland; ^6^ Department of Surgery Harvard Medical School, Massachusetts General Hospital Boston Massachusetts USA; ^7^ Department of Medicine University of Pittsburgh School of Medicine Pittsburgh Pennsylvania USA

**Keywords:** high‐resolution mass spectrometry (HRMS), melanoma cell‐derived exosomes (MTEX), proteomics, small extracellular vesicles (sEV), tumour‐derived exosomes (TEX)

## Abstract

Molecular profiling of small extracellular vesicles (sEV) isolated from plasma of cancer patients emerges as promising strategy for biomarkers discovery. We investigated the proteomic profiles of sEV immunoselected using anti‐CSPG4 antibodies from 15 melanoma patients’ plasma. The proteomes of sEV separated into melanoma cell‐derived (MTEX) and non‐malignant cell‐derived (NMTEX) were compared using high‐resolution mass spectrometry. Paired analysis identified the MTEX‐associated profile of 16 proteins that discriminated MTEX from NMETEX. We also identified the MTEX profile that discriminated between seven patients with no evidence of melanoma (NED) after therapy and eight with progressive disease (PD). Among 75 MTEX proteins overexpressed in PD patients, PDCD6IP (ALIX) had the highest discriminating value, while CNTN1 (contactin‐1) was upregulated only in MTEX of NED patients. This is the first report documenting that proteomes of tumour‐derived sEV in patients’ plasma discriminate cancer from non‐cancer and identify proteins with potential to serve as prognostic biomarkers in melanoma.

## 1 INTRODUCTION

Melanoma is among the most aggressive and therapy‐resistant human cancers. The epidemiology of melanoma is complex, and individual risk depends on the sun and other UV exposure, host genetic factors and their interactions (Shannan et al., 2016; Tripp et al., 2016). The successes of immunotherapy and targeted therapies have vastly changed the treatment and prognosis of melanoma in the last five years. Therapy with immune checkpoint inhibitors (ICIs) has induced long term responses and improved survival in a fraction of patients (Hodi et al., 2010). However, many patients still do not respond to ICIs for reasons that are not clear but are putatively related to the pre‐existing tumour‐induced immune suppression that is not overcome by ICIs. Tumour cell escape from the host immune system remains the major barrier to successful immunotherapy (Weiss et al., 2019). Numerous cellular and molecular mechanisms responsible for the dysregulation of tumour antigen‐specific immune responses in patients with cancer have been identified and studied in the last two decades (Whiteside et al., 2016; Yaguchi & Kawakami, 2016). Among these mechanisms, interactions between cancer cells and the tumour microenvironment (TME) are considered to be critically important for tumour progression (Cai et al., 2018; Whiteside, 2014).

Extracellular vesicles (EV) have recently emerged as an intercellular communication system that mediates the molecular cross‐talk between malignant and non‐malignant cells in the TME (Maas et al., 2017; Whiteside, 2017). EV are highly heterogeneous and include different vesicle classes varying in size, mechanisms of biogenesis, and molecular cargos (Willms et al., 2018). Small virus‐size EV (sEV) that originate from the endocytic compartment of cells are referred to as exosomes, while those produced by cancer cells are referred to as “tumour cell‐derived exosomes” (TEX). EV are involved in many aspects of cell‐to‐cell communication, including interactions between cancer and immune cells (Whiteside, 2017). We and others have reported that TEX play a key role in tumour‐induced suppression of immune effector cells and promote tumour growth by autocrine, juxtacrine, or paracrine mechanisms (Ruivo et al., 2017). It has been reported that the molecular content of exosomes mimics that of parent cells. Therefore, TEX could potentially serve as a “liquid biopsy” for non‐invasive tumour diagnosis or the assessment of prognosis and are currently of special interest.

Several attempts to characterize EV derived from different types of cancer cells have been made, including studies of TEX derived from cultures of melanoma cell lines (Valenti et al., 2007; Wieckowski et al., 2009). However, only a few studies characterizing TEX among total EV isolated from the blood of melanoma patients have been reported, and these studies highlight the biomarker potential of TEX (Peinado et al., 2012; Sharma et al., 2018). For example, the analysis of exosomes isolated from plasma of patients with stage IV melanoma reported increased levels of vesicular TYRP2, VLA‐4, and HSP70, while only TYRP2 levels were elevated in vesicles from the stage III disease when compared to healthy controls (Peinado et al., 2012). However, systematic proteomics analysis of sEV released in vivo from melanoma cells and isolated from the peripheral circulation of melanoma patients has not been reported so far.

The concept of a non‐invasive “liquid biopsy” of cancer includes sEV in the patients’ peripheral circulation. However, sEV are released into extracellular space by all cell types and, therefore, vesicles present in body fluids represent a heterogeneous mixture of different EV subpopulations. To demonstrate that TEX present in plasma (or other body fluids) of cancer patients can serve as a surrogate of tumour cells, it is necessary to separate TEX from vesicles produced by non‐malignant cells. We have recently described an immunocapture‐based method that separates melanoma cell‐derived TEX (MTEX) from sEV produced by non‐malignant cells (NMTEX) (Sharma et al., 2020). A pilot characterization of these exosome fractions using quantitative on‐bead flow cytometry and functional assays showed that MTEX were strongly immunosuppressive, but their molecular characterization was limited to a few selected markers related to immune reactivity (Sharma et al., 2020). Here, we have extended the molecular characterization of MTEX present in the plasma of melanoma patients using a comprehensive proteomics approach that allows for a deeper and broader analysis of proteins in MTEX and a better understanding of their molecular and functional significance.

## 2 MATERIALS AND METHODS

### 2.1 Patients

Blood samples were obtained from patients with melanoma treated at the UPMC Hillman Cancer Center Melanoma Program Outpatient Clinic by John M. Kirkwood, MD, and colleagues. Blood samples were collected for research under the University of Pittsburgh IRB approval #970186. All blood donors signed an informed consent form. The study included specimens collected from 15 melanoma patients (the disease status and clinicopathological information for all patients are listed in the Table S1). In addition, we collected blood specimens from five consented healthy donors (HDs) (IRB approval #04‐001) for proteomics analysis of total plasma exosomes. Blood samples were processed to separate plasma which was divided into aliquots and stored at −80°C until thawed and used for exosome isolation.

### 2.2 Total plasma exosome isolation

Exosomes were isolated from plasma of patients with melanoma or HDs by the mini‐SEC method optimized in our laboratory (Hong et al., 2016). Briefly, plasma samples stored at −80°C were thawed and centrifuged at 2000 x *g* for 10 min followed by another centrifugation at 10,000 x *g* for 30 min at 4°C. Samples were then ultra‐filtered through 0.22 μm filters (EMD Millipore, Billerica, MA). An aliquot (1 ml) of plasma was loaded onto a 10 cm‐long SEC column and 1 ml fractions were eluted with PBS. The void volume fraction #4 containing the majority of non‐aggregated, morphologically intact sEV was collected and used for analyses. Transmission electron microscopy (TEM), the vesicle size range, particle numbers, and protein content of fraction #4 were determined. The phenotype of vesicles was evaluated as previously described (Ludwig et al., 2019, Ludwig et al., 2019). The sEV protein concentration was determined by the BCA method (Pierce Biotechnology, Rockford, CA) as per manufacturer's instructions. sEV were concentrated using Vivaspin 500 (100,000 MWCO, Sartorius, Göttingen, Germany).

### 2.3 Immunoaffinity‐based separation of MTEX and NMTEX

MTEX (melanoma‐derived TEX) were separated from NMTEX using the immunoaffinity capture method as described by us earlier (Sharma et al., 2018; Sharma et al., 2020). Selection of the capture mAb, anti‐CSPG4, was based on the extensive analysis of its specificity for chondroitin sulfate peptidoglycan 4 (CSPG4), which selectively recognizes an epitope overexpressed on most (>80%) melanoma cells and melanoma stem cells but is not detectable in normal tissues, except for pericytes as previosly reviewed (Campoli et al., 2010; Ferrone & Whiteside, 2020). Anti‐CSPG4 mAbs (clones 763.64 or 225.28) were biotinylated using a one‐step antibody biotinylation kit (Novus Biologicals) following the manufacturer's protocol. An aliquot of sEV (10 μg protein) from fraction #4 was used for immunocapture on biotin‐labelled mAb‐charged streptavidin magnetic beads. Briefly, sEV were incubated with biotin‐labelled anti‐CSPG4 mAb overnight, then 100 μl of Streptavidin‐coated magnetic beads (washed twice with PBS) were added to the sEV‐mAb complex and incubated overnight. The recovered beads‐bound complexes were washed twice with PBS and re‐suspended in 250 μl of PBS as the MTEX fraction. The beads‐unbound material was stored as the NMTEX fraction. Detection of proteins in the MTEX and NMTEX cargo was performed by on‐bead flow cytometry.

### 2.4 Bead‐assisted flow cytometry

For the analysis of proteins carried on the surface or in the lumen of the isolated sEV, only the non‐immunocaptured total sEV and NMTEX subsets in solution could be used. MTEX captured on immunobeads could not be so tested. Aliquots of sEV (30 μg protein) were lysed using 1%Triton‐X100 for 10 min. The resulting sEV lysates were co‐incubated with 1 μl aliquots of aldehyde/sulfate latex beads (Thermo Fisher Scientific, #A37304, bead size 4 μm) for 1 h at room temperature with mild vortexing to load the lysate onto the beads. Then, the protein loaded beads were blocked with 2% (w/v) BSA for 1 h followed by washing with PBS. The beads were then incubated with primary antibodies (Anti‐Alix, #MA5‐32773, Thermo Fisher Scientific, clone JM85‐31, 1:100; Anti‐CSPG4, #AF2585, R&D Systems, clone LHM‐2, 1:100) for 1 h. The beads were then washed and stained with PE‐conjugated secondary antibody (1:100 dilution) for 30 min. Finally, the beads were washed with PBS and analysed by flow cytometry using Cytoflex S (Beckman Coulter).

### 2.5 Sample preparation for MS

sEV samples (MTEX and NMTEX fractions or total plasma sEV from patients or HDs) were mixed with a lysis buffer containing 4% (w/v) SDS, 100 mM Tris/HCl pH 8.0, 0.1 M DTT (buffer to sample volumetric ratio of 1:9), then boiled for 10 min and subsequently subjected to Filter‐Aided Sample Preparation (FASP) procedure (Wisniewski et al., 2009). Sequencing‐grade modified trypsin (Promega) was used at the enzyme to protein ratio of 1:100 (w/w); 50 mM ammonium bicarbonate was employed as a digestion buffer then incubation in a wet chamber was performed for 18 h at 37°C. The collected tryptic peptides were subsequently purified on C18 StageTips, each prepared by stacking six layers of Empore™ Octadecyl C18 extraction disk (3 M, Maplewood, MN, USA) in a 0.2 ml pipette tip. Peptide purification was performed by three consecutive washes with 5% methanol, 0.1% TFA (centrifugation at 4000 × *g*, 5 min) followed by additional two washes with 0.1% TFA. Elution was done using 60% ACN, 0.1% TFA. Eluates with purified peptides were evaporated to dryness in a vacuum centrifuge, peptides were reconstituted in 20 μl of LC‐MS grade water and subjected to peptide assay using tryptophan fluorescence method described by Wiśniewski and Gaugaz (Wisniewski & Gaugaz, 2015) (fluorescence measurement was conducted for the whole volume of each sample, i.e., 20 μl). After the measurement samples were acidified with TFA to achieve the final concentration of 0.1% (v/v) and subjected to LC‐MS/MS analysis.

### 2.6 Protein identification and quantitation by MS

The analysis was performed with the use of the Dionex UltiMate 3000 RSLC nanoLC System connected to the Q Exactive Plus Orbitrap mass spectrometer (Thermo Fisher Scientific). Peptides from each fraction (0.5 μg) were separated on a reverse‐phase Acclaim PepMap RSLC nanoViper C18 column (75 μm × 25 cm, 2 μm granulation) using acetonitrile gradient (from 4 to 60%, in 0.1% formic acid) at 30°C and a flow rate of 300 nL/min (total run time: 180 min). The spectrometer was operated in data‐dependent MS/MS mode with survey scans acquired at the resolution of 70,000 at m/z 200 in MS mode, and 17,500 at m/z 200 in MS2 mode. Spectra were recorded in the scanning range of 300–2000 m/z in the positive ion mode. Higher energy collisional dissociation (HCD) ion fragmentation was performed with normalized collision energies set to 25. Protein identification was performed using a reviewed Swiss‐Prot human database (release 2018_11_30 containing 11 378 269 sequence entries) with a precision tolerance 10 ppm for peptide masses and 0.02 Da for the fragment ion masses. All raw data obtained for each dataset were imported into Protein Discoverer v.1.4 (Thermo Fisher Scientific) < Thermo raw files > for protein identification and quantification (Sequest engine was used for database searches). Protein was considered as positively identified if at least two peptides per protein were found by the search engine, and a peptide score reached the significance threshold FDR = 0.01 (assessed by the Percolator algorithm); a protein was further considered as “present” if detected in at least one sample of a given type. The abundance of identified proteins was estimated in Proteome Discoverer using Precursor Ions Area detector node, which calculates the abundance of a given protein based on average intensity of three most intensive distinct peptides for this protein, with further normalization to the total ion current (TIC).

### 2.7 Western blots

sEV isolated from plasma, immunocaptured sEV or cell lysates (10 ug protein) were separated on 7–15% SDS/PAGE gels and transferred onto PVDF membrane (Millipore, Billerica, MA, USA) for western blot analysis. Membranes were incubated overnight at 4°C with antibodies specific for ALIX (PDCD6IP) (1:500, #2171S, Cell Signaling), Gelsolin (1:500, #MA5‐34684, Thermo Fisher Scientific), Contactin‐1 (1:250, #MAB9041, R&D Systems), and TSG101 (1:500, PA5‐31260, Thermo Fisher). Next, the HRP‐conjugated secondary antibody (1:10,000, Pierce, Thermo Fisher) was added for 1 h at room temperature (RT) and blots were developed with ECL detection reagents (GE Healthcare Biosciences, Pittsburgh, PA, USA). The intensities of the bands on exposed films were quantified using Image J software (NIH, USA).

### 2.8 Statistical analysis

Immunoglobulins were filtered from further analysis. To define proteins distinguishing MTEX from NMTEX, the set of identified proteins was split into two groups depending on the number of patients with observed measurements. The first group included proteins with non‐zero measurements observed for at least eight of 15 patients for MTEX or NMTEX samples. These proteins were analysed with the use of a non‐parametric one‐sided paired Wilcoxon test, testing the hypothesis of higher median protein abundance in MTEX samples when compared to NMTEX samples. The rank‐biserial coefficient of correlation (RBCC) for the Wilcoxon test (Kerby, 2014) was calculated as a measure of the effect size. The second group included the remaining proteins. These proteins were analysed regarding their presence‐absence status in each sample. The McNemar test for related measurements was applied with the support of Cohen's g for proportions as a measure of the effect size (Cohen & Hillsdale, 1988). *P*‐value for protein selection equal to 0.05 was considered the significance threshold (a functional analysis served as an additional false discovery verification). To define a panel of proteins discriminating melanoma patients with progressive disease (PD) from those with no evident/stable disease (NED/SD), U Mann‐Whitney test with corresponding Wendt effect size r_U_ (Wendt, 1972) was applied to the MTEX‐NMTEX difference levels. In the case of effect size measured by rank‐biserial coefficients of correlation (both RBCC and r_U_), the critical value for the large effect was set to 0.5. Cohen's g for proportions of at least 0.25 were interpreted as indicating the large effect (Cohen & Hillsdale, 1988). The effect size quantification was done for the absolute values of the relevant statistics. Large effect size values indicate differences with a very high level of confidence. Next, the decision tree classifier with five fold cross‐validation was applied to find the protein signature differentiating melanoma patients with PD from those with NED/SD. A set of classifiers was constructed in a stepwise procedure to rank the proteins according to their informativeness.

### 2.9 Bioinformatics analysis

A list of genes corresponding to differentially expressed proteins was used to search for enriched Gene Ontology terms and Reactome pathways by Fisher test. Bioconductor packages ReactomePA (Yu & He, 2016) and clusterProfiler (Yu et al., 2012) were used. To minimize false discoveries, terms and pathways with at least three and at most 600 genes assigned to them were tested only. The whole human genome served as a reference for the enrichment analysis of all proteins present in MTEX. For the remaining enrichment analyses, genes corresponding to all identified proteins (i.e., 573 proteins) served as a reference. FDR for tested GO terms and Reactome pathways was estimated with the Storey method. The threshold for q‐value was set at 0.05. String‐db database (Szklarczyk et al., 2019) was used to predict relations between chosen proteins.

### 2.10 Data storage and availability

The HRMS‐based proteomic data have been deposited to the ProteomeXchange Consortium via the PRIDE (https://www.ebi.ac.uk/pride) (Deutsch et al., 2020; Perez‐Riverol et al., 2019) partner repository with the dataset identifier PXD021285 and PXD022867.

## 3 RESULTS

### 3.1 Characteristics of sEV isolated from plasma of melanoma patients or healthy donors

**FIGURE 1:**
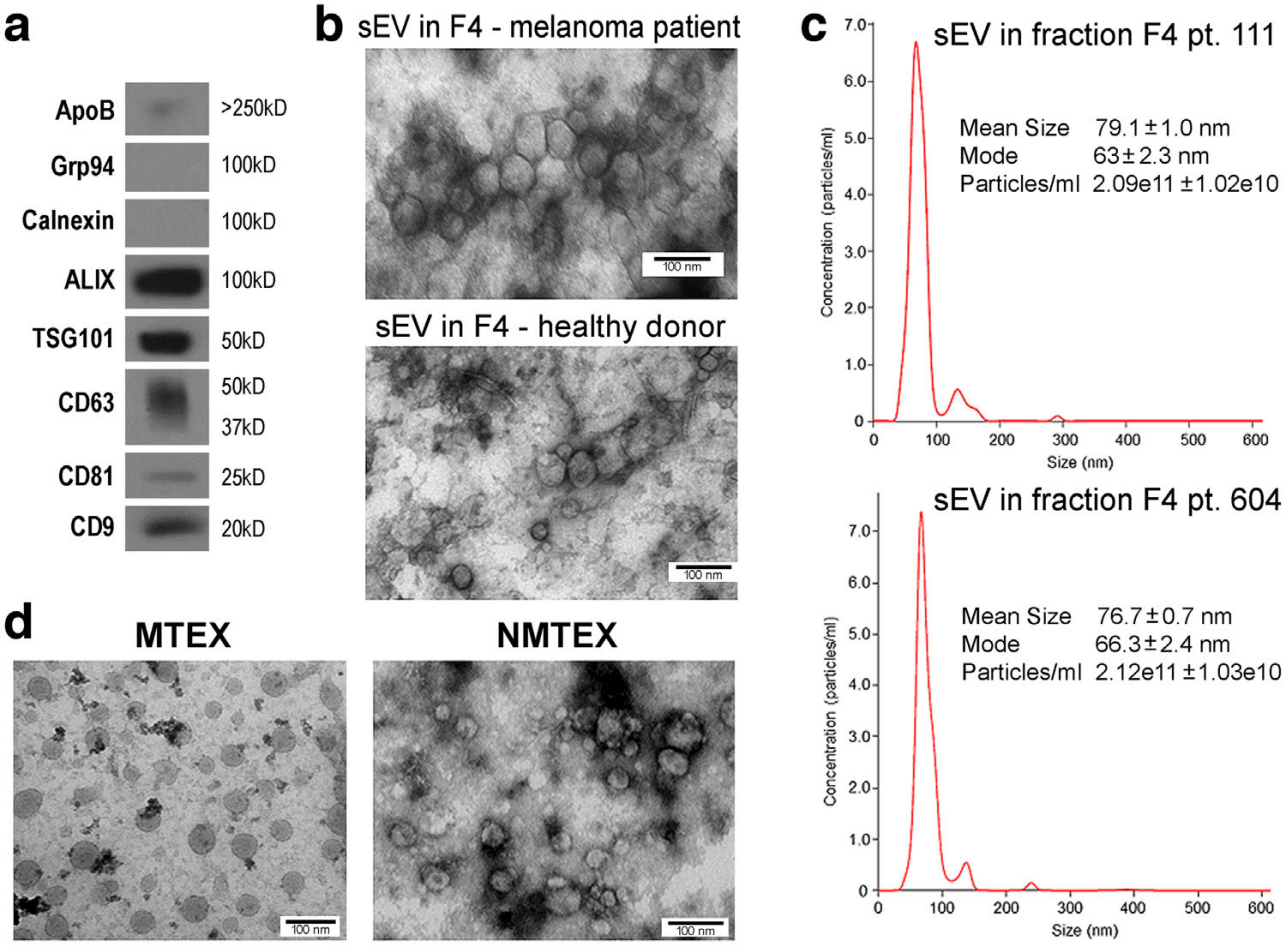
Characterization of sEV isolated from plasma. Panel a – western blot characterization of exosome markers in sEV collected in fraction #4 as described in Materials and Methods. Panel b – a TEM image of sEV in fraction #4 obtained from plasma of a melanoma patient or healthy donor. Panel c – NanoSight profiles of EVs in fraction #4 of two patients with melanoma. Panel d – TEM of MTEX detached from anti‐CSPG4 mAbs on beads and NMTEX that remain in suspension following immune capture

In this study, sEV isolation by SEC was performed using pre‐cleared, ultrafiltered plasma specimens of 15 patients with metastatic melanoma. EVs collected in fraction #4 were used for immunocapture of MTEX as previously reported (Sharma et al., 2018; Sharma et al., 2020). Figure 1a shows western blots of isolated EVs in fraction #4 which carry ALIX, TSG101 and tetraspanins, CD63, CD81 and CD9, but not cytoplasmic proteins such as calnexin or Grp94. The content of ApoB is minimal. Figure 1b illustrates TEM images of EVs in fraction #4 isolated from plasma of a patient with melanoma or from plasma of a healthy donor (HD) indicating comparable vesicular morphology and size. Figure 1c shows representative NanoSight profiles for EVs in fraction #4 for two melanoma patients. The size (∼80 nm), vesicular morphology by TME and endosomal origin in WBs suggest that these EVs fit in the category of small EVs and are designated as “**sEV**” in this study.

The sEV in fraction #4 of all 15 patients carried different relative levels of the CSPG4 epitope on the vesicle surface by quantitative on‐bead flow cytometry performed as previously described (Sharma et al., 2018; Sharma et al., 2020) and as presented in Figure S1. These sEV were separated into MTEX and NMTEX by immune capture using anti‐CSPG4 mAb. Figure 1d shows representative TEM images of MTEX and NMTEX obtained by immune capture. MTEX, which were detached from anti‐CSPG4 mAb on beads by a brief exposure to pH 2.5 buffer followed by neutralization, appear as slightly larger and “cleaner” vesicles. The MTEX and NMTEX separated by immune capture were used for paired comparative proteomic analysis. The total plasma sEV of five HDs underwent the same proteomic analysis. However, as HDs do not have MTEX, the available data do not contribute to the paired MTEX/NMTEX analysis and, therefore, are not shown. Nevertheless, preliminary proteomic analysis of HD's EVs in fraction #4 showed that 282 proteins were shared with NMTEX, while 75 proteins were upregulated in NMTEX. These data suggest that the proteomic profiles of NMTEX and sEV isolated from plasma of HDs are partly distinct, and that the biological significance of these differences deserves to be independently evaluated in future studies.

### 3.2 Proteins detected in MTEX

The levels of total sEV protein (TEP) varied between melanoma patients from 54 to 92 μg/ml plasma (Table S1). Importantly, the average TEP level at 63 μg/ml for patients with non‐evident or stable disease (NED/SD; *n* = 7) at the time of blood draw was lower than that for melanoma patients with progressive disease (PD; *n* = 8) at 78 μg/ml (*P* < 0.02). The ratios of MTEX/TEP varied, ranging from 0.32 to 0.75, while those for MTEX/NMTEX varied from 0.48 to 3.05 (Table S1), and these ratios did not reflect disease activity. Noteworthy, as documented in Figure S2, MTEX were enriched in melanoma‐associated antigens (MAAs), including CSPG4, Melan A, Gp100 and VLA4, while the corresponding NMTEX samples were negative, confirming our previously reported data (Sharma et al., 2020).

Paired MTEX and NMTEX samples of 15 patients were analysed using a shotgun proteomics approach based on the high‐resolution mass spectrometry (HRMS). This approach allowed for the identification of about 800 proteins. Further analyses identified 573 proteins encoded by the unique genes (immunoglobulins and putative uncharacterized proteins were excluded). The complete list of proteins identified and quantified in 30 exosome specimens (15 MTEX + 15 NMTEX) is presented in Table S2, while the abundance values for each protein in the analysed samples are presented as a heatmap in Figure S3. Interestingly, this heatmap indicates that in eight patients with PD, the level of many proteins was substantially higher than that in seven patients with NED/SD. This was especially striking for patients 7, 8, 9, and 10 as listed in Table S1.

### 3.3 Proteome components characteristic for MTEX

To identify proteins with significantly upregulated levels in MTEX, that is, proteins which levels discriminated MTEX and NMTEX, the ratios of individual protein levels in MTEX and NMTEX were determined for each patient. There were 384 proteins detected in the samples obtained from more than half of the included patients (8/15; representing the “continuous” mode of the statistical testing). These included 62 proteins that were upregulated in MTEX (*P*‐value > 0.05 and RBCC ≥0.5; Table S2). Furthermore, when the remaining 189 proteins were subjected to the binary mode of analysis (i.e., using the absent/present algorithm), there were 11 additional proteins that were found to be upregulated in MTEX (*P*‐value > 0.05 and Cohen g ≥0.5; Table S2). Hence, 73 protein species (62+11) in the patients’ plasma represented a subset of proteins upregulated in MTEX compared to NMTEX. Next, to identify proteins with levels that were markedly lower in MTEX than in NMTEX, effect size values were considered. Because all MTEX‐upregulated proteins showed large effect size, the same threshold was required to call MTEX‐downregulated proteins. Consequently, 77 proteins were classified as downregulated in MTEX (effect size ≤−0.5; Table S2). The Venn diagram in Figure 1a shows that 496 sEV proteins were detected in MTEX, including 73 proteins that were markedly upregulated and 77 proteins that were significantly downregulated.

**FIGURE 2:**
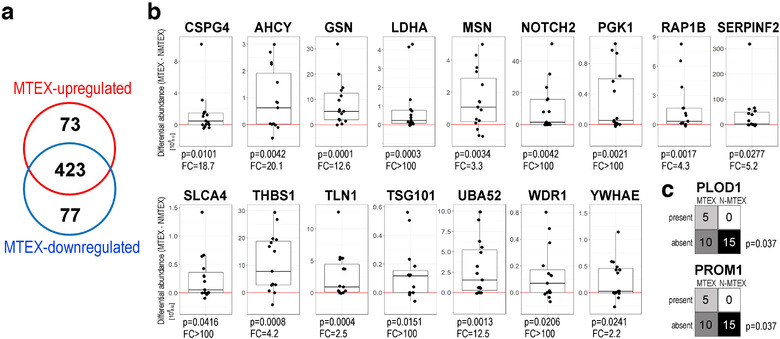
MTEX proteins found to have significantly upregulated levels relative to NMTEX. Panel a – the Venn diagram showing numbers of proteins upregulated or downregulated in MTEX. Panel b – individual differences in the protein levels between paired samples of MTEX and NMTEX; boxplots show median, upper and lower quartile, maximum and minimum (dots represent individual patients; the red line represents no difference between MTEX and NMTEX, FC – average fold‐change). Panel c – PLOD1 and PROM 1 are shown as examples of the present/absent status analysis of proteins in paired samples of MTEX and NMTEX

Among the 73 proteins that were significantly increased in abundance in MTEX, we were especially interested in those known to be involved in cancer progression as well as those proteins that were detected in more than 8/15 MTEX samples we examined. Figure 2b lists sixteen such proteins: AHCY, LDHA, GSN, NOTCH2, THBS1, UBA52, TLN1, PGK1, SERPINF2, WDR1, CSGP4, MSN, SLC1A4, YWHAE, TSG101, and RAP1B. Differences in levels of these proteins in paired MTEX and NMTEX samples of individual patients are presented. Furthermore, two additional proteins, PLOD1 and PROM1, that were detected only in a smaller proportion of melanoma specimens (and thus exemplify proteins in the characteristic absent/present mode) are illustrated in Figure 2c. The group of 16 selected MTEX proteins discriminated MTEX from NMTEX and could potentially be useful in differential analysis of plasma sEV in patients with melanoma. Noteworthy, the CSPG4 antigen used for the MTEX immunocapture was detected by LC‐MS/MS in all MTEX specimens, and its median upregulation compared to NMTEX was about 19‐fold. In addition to CSPG4, a few MTEX‐upregulated proteins identified by LC‐MS/MS, including PDCD6IP (ALIX), Gelsolin (GSN), and contactin‐1 (CNTN1) were further analysed by the immune‐based methods, which confirmed their reduced levels in NMTEX (Figure S4).

### 3.4 Biological functions associated with proteins characteristic for MTEX

To identify biological pathways associated with the proteins detected in MTEX, the analysis of gene ontology was performed after their annotation with the coding genes. First, pathways associated with 496 proteins abundant in MTEX (i.e., excluding 77 proteins downregulated in MTEX) were analysed, using the whole human genome as the reference. There were numerous significantly overrepresented GO terms associated with proteins present in MTEX listed in Table S3. These included “extracellular structure organization” (81 proteins), “wound healing” (87 proteins), “regulation of vesicle‐mediated transport” (54 proteins), “acute inflammatory response” (44 proteins), and “protein activation cascade” (46 proteins). Moreover, 39 MTEX proteins were associated with the GO term “melanosome” (GO:0042470), out of 106 proteins listed in the human genome reference.

**FIGURE 3:**
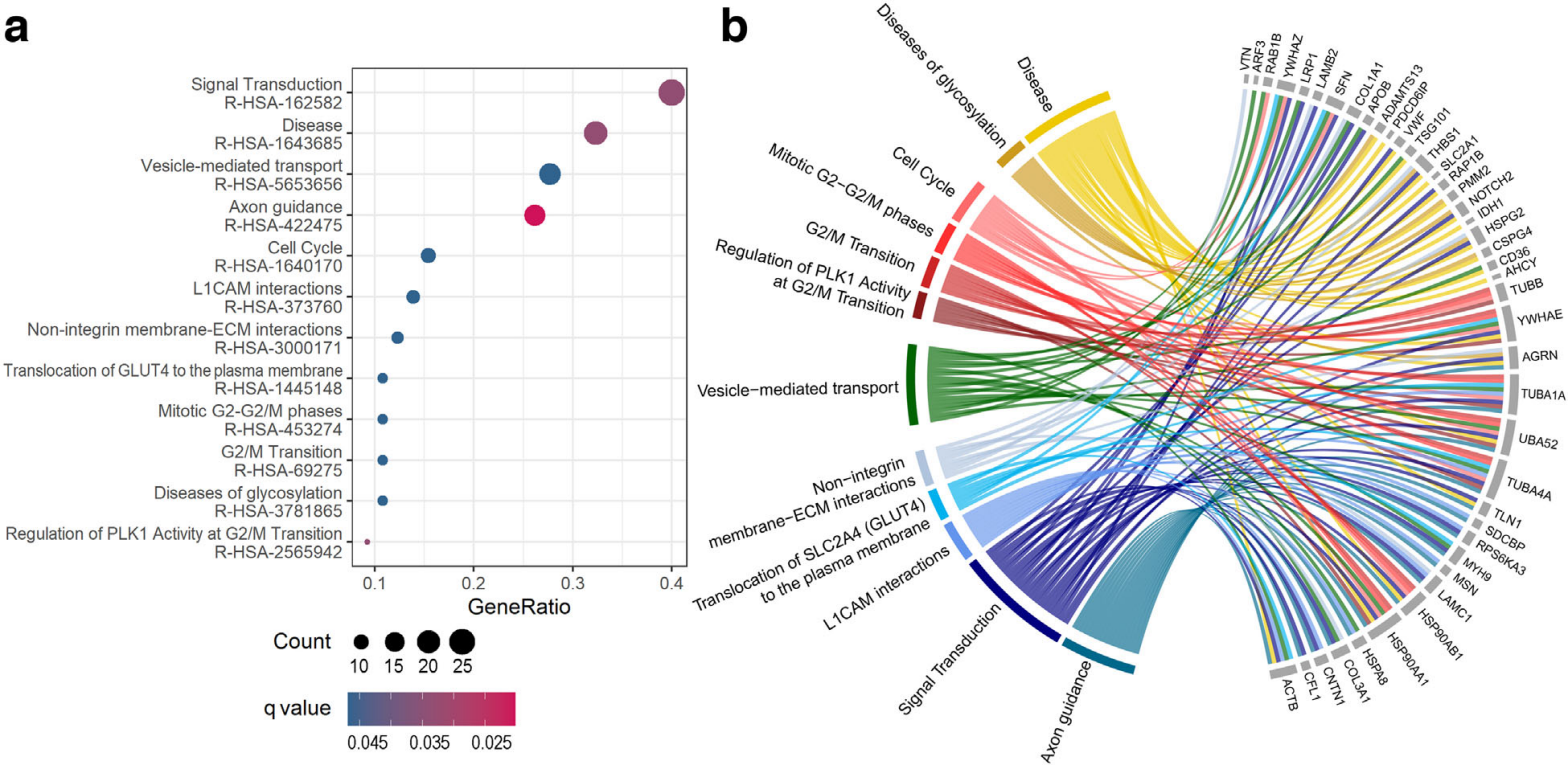
Reactome pathways analysis. Panel a shows a dot‐plot with significantly enriched Reactome pathways (q < 0.05) coloured by FDR. GeneRatio on x‐axis refers to the ratio of the number of differentially expressed genes in a pathway (Count) to the number of all differentially expressed genes (*n* = 73). The size of each dot corresponds to the number of differentially expressed genes in a pathway. Panel b shows a network of genes linked to the Reactome pathway terms

Next, the more specific analysis of gene ontologies was performed for the 73 proteins upregulated in MTEX (the list of 573 detected proteins was used as the reference). There were 393 GO terms associated with MTEX‐upregulated proteins that were overrepresented (*P* < 0.05), yet only the establishment of cell polarity (GO:0030010) remained statistically significant after the multiple testing correction (q < 0.05; Table S4). The analysis of functional interactions between the MTEX‐upregulated proteins was performed using the Reactome database (Jassal et al., 2020). This analysis enabled detection of 36 pathways that were significantly enriched (*P* < 0.05; see Table S5). Of these 36 pathways, twelve remained statistically significant after the FDR correction (q < 0.05); these were pathways involved in signal transduction, cell cycle progression, cell adhesion, and protein glycosylation. These overrepresented pathways and their corresponding protein components detected in MTEX are presented in Figure 3.

**FIGURE 4:**
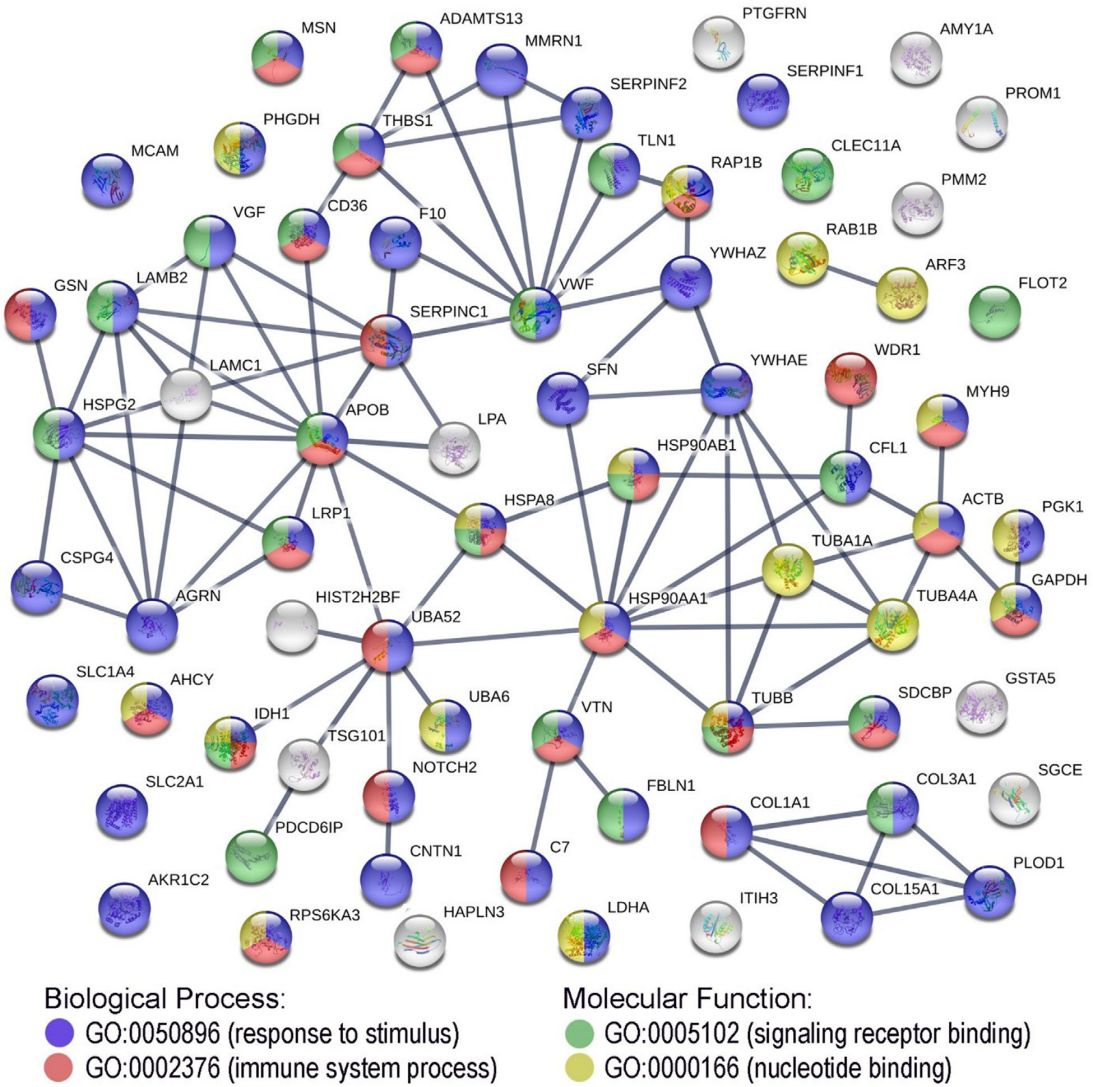
An interaction map for proteins found to be upregulated in MTEX. Proteins associated with the four selected GO terms are colour‐coded

Furthermore, to illustrate possible interactions among all 73 proteins upregulated in MTEX, an additional analysis was performed using the String‐db database (Szklarczyk et al., 2019). Potential interactions among these proteins are presented in Figure 4. The most numerous GO term associated with these proteins was “response to stimulus” (43 proteins), while connected molecular functions “signaling receptor binding” and “nucleotide binding” were attributed to 23 and 19 proteins, respectively. In aggregate, the above‐presented data indicated that MTEX‐associated proteins are mainly involved in signal transduction. Moreover, there were 28 proteins associated with the term “immune system process”, which suggested that many MTEX‐associated proteins mediate immune regulatory functions (all above‐mentioned terms were statistically overrepresented, although the whole human genome was used as a reference in the String‐db database, which made the statistical testing less reliable).

In addition to the “unsupervised” analysis of processes associated with the proteins upregulated in MTEX as described above, we specifically searched for proteins known to be expressed in melanoma. Taking advantage of the TCGA database (The TCGA Research Network), we confirmed that transcripts for the vast majority of MTEX‐upregulated proteins were listed in the TCGA melanoma database (Table S6). Further, more than half of MTEX‐upregulated proteins (38 out of 73) were present at high or moderate levels in melanoma tissues according to immunohistopathology data available in the Protein Atlas (Table S6) (The Human Protein Atlas). Moreover, 10 of MTEX‐upregulated proteins are present in the melanosome (GO:0042470). There were also 28 MTEX‐upregulated proteins associated with immune‐related functions (GO:0002376), several of which are not expressed in melanoma tissues listed in the Protein Atlas (Table S6). Nevertheless, some of these upregulated proteins are known to possess immunoregulatory activity in melanoma (e.g., contactin1, fibulin, isocitrate dehydrogenase).

### 3.5 MTEX proteins discriminate patients with progressing melanoma (PD) from those with no evident or stable disease (NED/SD) after therapy

**FIGURE 5:**
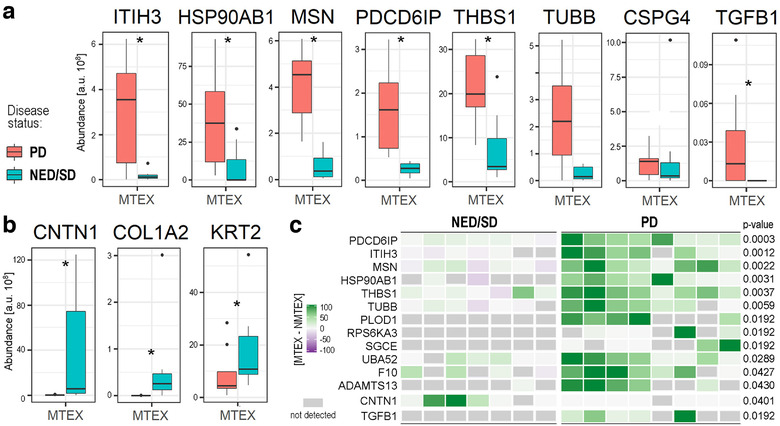
sEV‐associated proteins that were present at a significantly different level in MTEX isolated from plasma of melanoma patients with PD relative to MTEX isolated from plasma of melanoma patients with NED/SD. Panel a – MTEX proteins with significantly higher levels in patients with PD. Panel b – MTEX proteins with significantly higher levels in patients with NED/SD. Boxplots show median, upper and lower quartile, maximum, and minimum; dots represent outliers. The statistical significance of differences between patient subgroups (*P* < 0.05) is marked with asterisks. Panel c – A heat map presenting the differential (MTEX‐NMTEX) protein levels in individual melanoma patients with NED/SD (*n* = 7) or PD (*n* = 8). Twelve proteins found to be upregulated in MTEX of melanoma patients with PD are listed. Moreover, the levels of CNTN1 and TGFβ1 are presented in the corresponding samples. The relative levels of the listed proteins is colour‐coded where gray boxes represent not detected proteins; *P*‐values represent the significance of the difference between patients’ subgroups of the differential (MTEX‐NMTEX) value

Among 15 patients with metastatic melanoma (MM) donating plasma for this study, seven patients had NED/SD and eight had PD at the time of the blood draw for sEV recovery. All patients were previously treated for MM. To determine whether MTEX could confirm disease activity, the MTEX protein contents of the two patient groups were compared (see the heat map in Figure S3). We detected 83 proteins in MTEX that significantly differed in the two patient groups. There were 75 proteins whose differential (MTEX‐NMTEX) level was markedly higher in MTEX from patients with PD than in patients with NED/SD (Table S2), including 12 proteins with significantly upregulated MTEX relative to NMTEX, namely: PDCD6IP, HSP90AB1, ITIH3, MSN, THBS1, TUBB, UBA52, F10, PLOD1, RPS6KA3, SGCE, ADAMTS13 (the data for eight of these proteins are shown in Figure 5a). On the other hand, there were eight proteins with a significantly lower level in MTEX from patients with PD than in those with NED/SD. The data for three of these proteins, including CNTM1 (contactin1, the only protein consistently upregulated in MTEX of NED/SD patients), are shown in Figure 5b.

Among the 12 MTEX‐upregulated proteins with higher expression in PD patients, PDCD6IP (ALIX, ALG 2‐interacting protein X) discriminated best (*P* = 0.0003) between the two groups of melanoma patients (Figure 5c). Remarkably, the level of this protein alone was sufficient to allow for errorless discrimination of seven patients with NED/SD from eight patients with PD. In contrast, any discrimination attempts using a decision tree classifier based on the level of the remaining 11 proteins generated data with less discriminating power. Despite the small size of the patient groups, the data suggest that PDC6IP alone can serve as a potentially reliable biomarker able to discriminate melanoma patients with a different disease status. Furthermore, increased levels of CNTM1 in MTEX of NED/SD patients and its absence in MTEX of patients with PD identified another potential indicator of disease activity. Also, the absence of TGF‐β1 in MTEX of NED/SD patients was a significant discriminator of the two patient groups that together with the absence of CNTM1 in MTEX of patients with PD enhanced the prognostic value of this differential proteomic analysis.

**FIGURE 6:**
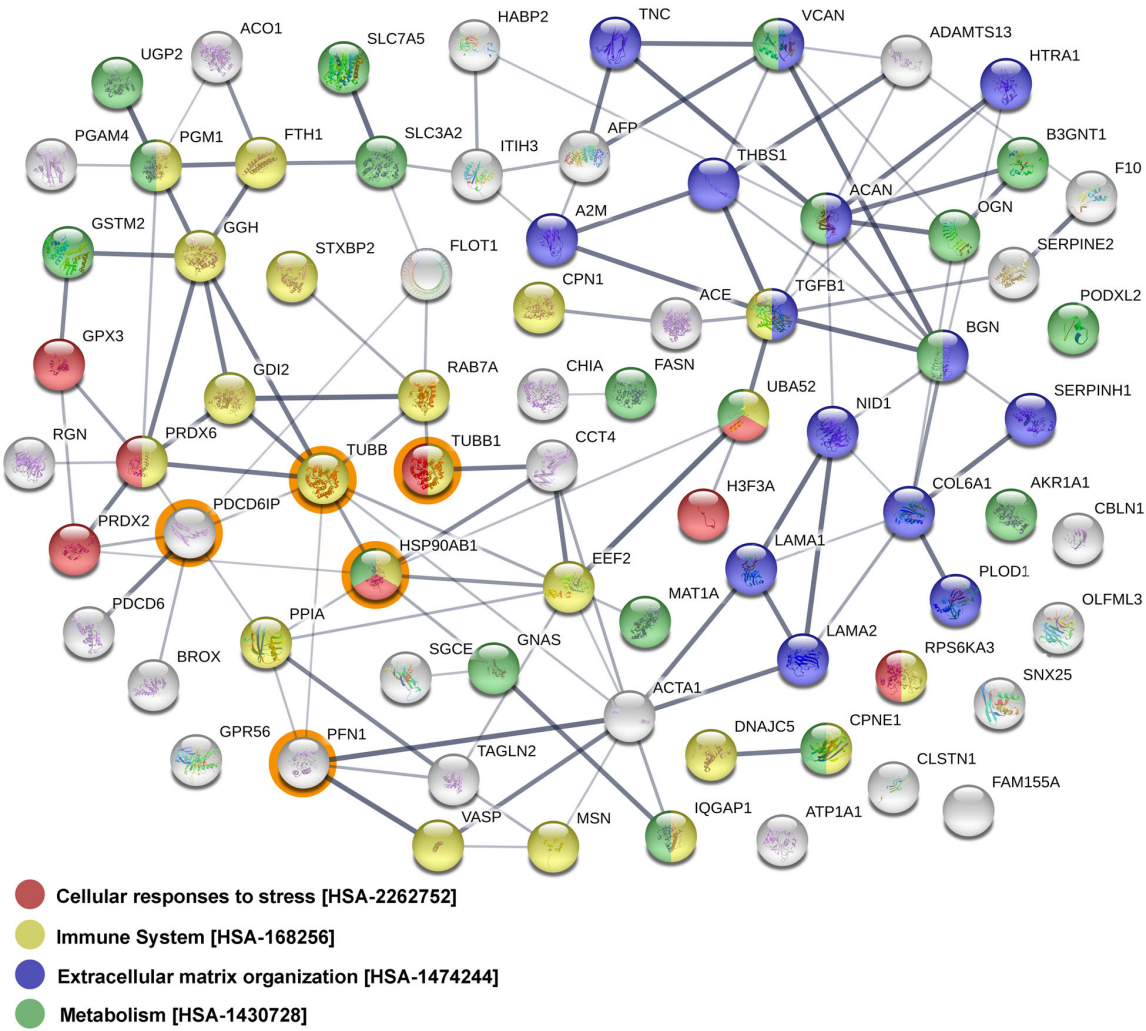
The network of potential interactions between MTEX proteins found to be overexpressed in patients with PD but not in patients with NED/SD. The four proteins (HSP90AB1, PFN1, TUBB, and TUBB1) strongly associated with PDCD6IP (ALIX) are circled in orange

A search for biological activities associated with 83 MTEX proteins (75+8) differentially expressed in the two groups of melanoma patients identified nine Reactome pathways at *P* < 0.05; yet none of them remained statistically significant after the multiple testing correction); see Table S7. The enriched pathways included processes involved in an extracellular matrix organization (15 proteins), metabolism (20 proteins, including nine proteins involved in the metabolism of carbohydrates), and cellular responses to stress (8 proteins). Moreover, there were 24 proteins involved in the immune system among them. The network of MTEX proteins characteristic for melanoma patients with a progressing disease (PD) is illustrated in Figure 6. Based on correlation analysis between PDC6IP and 83 MTEX proteins differentiating melanoma patients with PD from those with NED/SD, we found four proteins that strongly (r_u _> 0.7, *P* < 0.005) associated with PDC6IP, namely HSP90AB1, PFN1, TUBB, and TUBB1 (Figure S5).

## 4 DISCUSSION

The search for biomarkers of melanoma progression and response to therapy including recent genomics or other “omics” approaches has led to the discovery of numerous promising proteins (reviewed in (Gowda et al., 2020; Rodriguez‐Cerdeira et al., 2018)). However, none of these potential biomarkers have been validated so far, and only soluble lactic dehydrogenase (sLDH) remains as the protein that correlates with the disease burden in some patients with metastatic melanoma (Byström et al., 2017). Recent attempts at establishing correlations between levels of sLDH and any specific molecular, immunological or metabolic phenotypes, including immune cell infiltrate in the tumour, point mutations, DNA copy number, promoter methylation, RNA expression or protein expression in melanoma metastases have been not been successful (Gowda et al., 2020). Therefore, the search for biomarkers predictive of response to immune therapies remains an unmet clinical urgent need. sEV have emerged as a new potentially diagnostic/prognostic tool in melanoma. Factors known to be involved in angiogenesis, immune suppression, modification of stroma, capture of cancer cells in lymph nodes and tumour cell progression have been identified in sEV from the plasma of melanoma patients by us and others (Alegre et al., 2016; Gowda et al., 2020; Hood, 2019; Sharma et al., 2020). Several previous studies suggested that the use of EV, especially tumour cell‐derived sEV (TEX), might be a more promising approach to the discovery and development of melanoma‐associated biomarkers than strategies dependent on conventional tumour tissue examination or on measuring levels of soluble factors in patients’ plasma (Byström et al., 2017; Rodriguez‐Cerdeira et al., 2018; The TCGA Research Network).

Most of the earlier studies with melanoma cell‐derived TEX were performed using EV derived from melanoma cell lines. The most comprehensive proteomics profiling of EV proteins released by a panel of melanoma cell lines (1205Lu, 501MEL, A375M, Daju, G1, MNT‐1, SK‐MEL‐28) identified 917 proteins in total, with each cell line representing subsets of between 486 and 632 of these proteins (Lazar et al., 2015). A quarter of the identified proteins were common among the cell lines (e.g., ESCRT proteins, CD9, CD63, CD81, small GTP‐binding proteins, annexins, cytoskeletal, and motor proteins). There were 22 proteins specific for MTEX from non‐tumorigenic cell lines, 29 proteins specific for MTEX from tumorigenic cell lines, and 112 proteins unique of MTEX from metastatic cell lines. Proteins unique of MTEX from metastatic cell lines included EGFR, EPHB2, KIT, LGALS1/LEG1, LGALS3/LEG3, MCAM/MUC18, MET, NRAS, NT5E/5NTD/CD73, PTK2/FAK1, and SRC (Lazar et al., 2015). More recently, plasma sEV carrying PD‐L1 (EXO‐PD‐L1) have been used for monitoring melanoma patients treated with ICIs (Chen et al., 2018; Cordonnier et al., 2020). In agreement with correlative data reported for other solid tumours, the latter studies in melanoma showed that tracking changes in circulating levels of EXO‐PD‐L1 was associated with disease activity and progression (Ricklefs et al., 2018; Theodoraki et al., 2018) and predicted response to immune therapy (Chen et al., 2018; Cordonnier et al., 2020). While these results place EXO‐PD‐L1 in a category of promising biomarkers for melanoma and other cancer types, the tumour origin of EXO‐PD‐L1 remains speculative, as they were not validated TEX and might have largely originated from non‐malignant PD‐L1+ cells.

The objective of the present study was to determine whether MTEX obtained from plasma of patients with melanoma have the potential to serve as a non‐invasive liquid biopsy able to predict disease progression or response to therapy. The rationale for the current approach of comparing proteomic profiles of MTEX and NMTEX was based on an assumption that MTEX will serve as a more specific liquid biopsy of the tumour than total plasma sEV. Taking advantage of the previously developed immune capture of MTEX using anti‐CSPG4 mAbs (Sharma et al., 2018; Sharma et al., 2020), we separated MTEX from vesicles derived from non‐malignant cells (NMTEX) (Sharma et al., 2020). To evaluate MTEX as a potential liquid tumour biopsy, the LC‐MS/MS analysis of the protein cargos in paired MTEX and NMTEX was performed. The expectation, based in part on our earlier flow‐cytometry‐based analysis of MTEX and NMTEX (Sharma et al., 2020), was that a panel of proteins uniquely and consistently identifiable in MTEX would provide the signature for MTEX in support of their role as surrogates of melanoma progression or response to therapy.

We first determined that HRMS of paired MTEX and NMTEX specimens of randomly selected patients with metastatic melanoma (MM) identified a set of 73 proteins specific or overexpressed in MTEX. Based on our previous study, where the separation of MTEX from NMTEX showed clinical relevance (Sharma et al., 2020), we expected that the identified 73 proteins might be useful in discriminating MTEX from NMTEX. We selected a group of 16/73 MTEX proteins to serve as an “MTEX differentiating panel”. The selection was based on the criteria that included the known role of each protein in cancer progression, the presence of each protein in at least 8/15 MTEX examined, the known association with exosome membranes, and the inclusion in the Exocarta database (Keerthikumar et al., 2016). The protein‐associated pathways identified by GO analysis in the group of 73 proteins upregulated in MTEX showed interesting functional features. In agreement with our previously reported data (Sharma et al., 2020), MTEX were predominantly enriched in proteins engaged in signalling pathways and immunoregulatory activity. The previously reported functional distinction between MTEX and NMTEX using flow cytometry and immune response results (Sharma et al., 2020) was thus confirmed by HRMS of separated MTEX and NMTEX.

Perhaps more importantly, this study also identified a set of MTEX proteins, including ADAMTS13, CNTN1, F10, HSP90AB1, ITIH3, MSN, PDCD6IP, PLOD1, RPS6KA3, SGCE, THBS1, TUBB, and UBA52, whose levels discriminated patients with MM who had PD from those who were NED/SD after therapy. This set of proteins enabled us to propose the hypothetical MTEX‐based “prognostic signature”. Even with a very small number of MM patients in each group, it was possible to show that the 83 proteins differentially expressed in MTEX included those associated with the ECM organization, metabolism, responses to stress, and immune regulation. This finding suggests that MTEX have a protein profile that reflects melanoma progression and outcome. Interestingly, a recently reported proteomic analysis of EVs from exudative serome obtained after lymphadenectomy in patients with melanoma by Peinado's group also showed enrichment in proteins correlating with or recapitulating melanoma progression (García‐Silva et al., 2019).

Remarkably, despite the small patient numbers in each cohort, PDCD6IP (ALIX) emerged as the protein with the greatest power for discriminating melanoma patients with PD from patients with NED/SD. PDCD6IP (Programmed cell death 6‐interacting protein also known as ALIX, ALG2 interacting protein X, AIPI, KIAA1375) is a multifunctional protein involved in endocytosis, multivesicular body (MVB) biogenesis, membrane repair, cytokinesis, apoptosis and maintenance of tight junction integrity (Monypenny et al., 2018; Odorizzi, 2006). ALIX/PDCD6IP is best known as a component of the endosomal‐sorting complex required for transport (ESCRT) (Henne et al., 2011) involved in the concentration and sorting of cargo proteins directed to the MVB for incorporation into intraluminal vesicles (ILVs). More recent studies report that ALIX/PDCD6IP plays a role in tumour cell apoptosis and proliferation, regulates tumour‐mediated immunosuppression and controls PD‐L1 expression (García‐Silva et al., 2019). We found that 4/83 proteins differentiating PD from NED/SD patients strongly correlated with ALIX/PDCD6IP; these are HSP90AB1 (heat shock protein 90), TUBB (β‐tubulin), TUBB1 (Tubulin β1 chain) and PFN1 (profilin1). These proteins interact directly with each other, putatively forming the functional network. Moreover, CNTN1, a cell adhesion protein and a member of the immunoglobulin superfamily known to be expressed in melanoma (Deutsch et al., 2020; Jassal et al., 2020), was highly upregulated in MTEX of some patients with NED/SD and was not detectable in MTEX of PD patients. In contrast, TGF‐β1, which was not detected in MTEX of NED/SD patients, was overexpressed in MTEX of patients with PD.

Hence, the molecular signature of MTEX consisting of PDCD6IP/ALIX, 4 correlated proteins (HSP90AB1, TUBB, TUBB1, and PFN1) highly expressed in MTEX of patients with PD, plus CNTN1 and TGF‐β1 with differential distribution in MTEX of PD vs NED/SD patients may have prognostic significance in melanoma. Importantly, all these proteins have been reported to play a key role in melanoma progression and metastasis (Bracalente et al., 2016; Subramanian et al., 2004). In aggregate, our data indicate that the protein cargo of MTEX reflects the content of tumour cells, might serve as a liquid tumour biopsy and, upon further validation, it has a potential to become a surrogate of melanoma progression.

## Supporting information

Supplementary Figure S1. On‐bead flow cytometry for sEV in fraction #4 isolated from plasma of each melanoma patient included in this study (n = 15). sEV were immunocaptured on streptavidin beads using biotin‐labeled anti‐CD63 mAb as previously described (Sharma et al., 2018). Detection was performed using PE‐labeled anti‐CSPG4 mAb. Relative fluorescence intensity (RFI) values differ among patients but CSPG4+ sEV are present in total plasma‐derived vesicles of all 15 patients.

Supplementary Figure S2. On‐bead flow cytometry for detection of melanoma‐associated antigens (MAAs) on the surface of MTEX or NMTEX. Relative fluorescent intensity (RFI) values for each antigen are marked. MTEX are enriched in MAA relative to NMTEX as previously reported in (Sharma et al., 2020).

Supplementary Figure S3. The heat map representing the abundance of 573 proteins detected in MTEX and NMTEX samples of 15 MM patients. NED/SD patients and PD are grouped. Names of MTEX‐upregulated and MTEX‐downregulated proteins are highlighted in red and navy blue, respectively. The abundance of proteins is colour‐coded according to deciles of all normalized signals.

Supplementary Figure S4. The detection of selected MTEX‐upregulated proteins in sEV from melanoma. Panel a – quantitation of CSPG4 and PDCDIP (ALIX) relative levels by the on‐latex bead flow cytometry in the mixture of MTEX and NMTEX as well as NMTEX alone isolated from plasma of a MM patient; marked is relative fluorescence intensity (RFI) including isotype control (Ig‐PE). Panel b – Western blot analysis of ALIX, gelsolin (GSN), contactin‐1 (CNTN1), and TSG101 in sEV isolated from plasma of a MM patient and supernatant of Mel526 melanoma cell line; represented is the mixture of MTEX and NMTEX (Total) as well as NMTEX alone.

Supplementary Figure S5. The correlation between MTEX proteins discriminating MM patients with NED/SD and PD. Panel a – the correlation tree of 83 MTEX proteins which differential (MTEX‐NMTEX) level was significantly different (p < 0.05) between NED/SD and PD; proteins which level was highly correlated with the level of PDCDI6P are marked with orange dots and presented in the insert. Panel b – the pairwise correlation between PDCDI6P and 4 proteins: PFN1, HSP90AB1, TUBB, and TUBB1).

Tables S1‐S7
