# Supplementary figures and images for: Proteomic profile of melanoma cell‐derived small extracellular vesicles in patients’ plasma: a potential correlate of melanoma progression
Source: J Extracell Vesicles. 2021 Feb 11;10(4):e12063. doi: 10.1002/jev2.12063 (PMC7876545; doi:10.1002/jev2.12063)

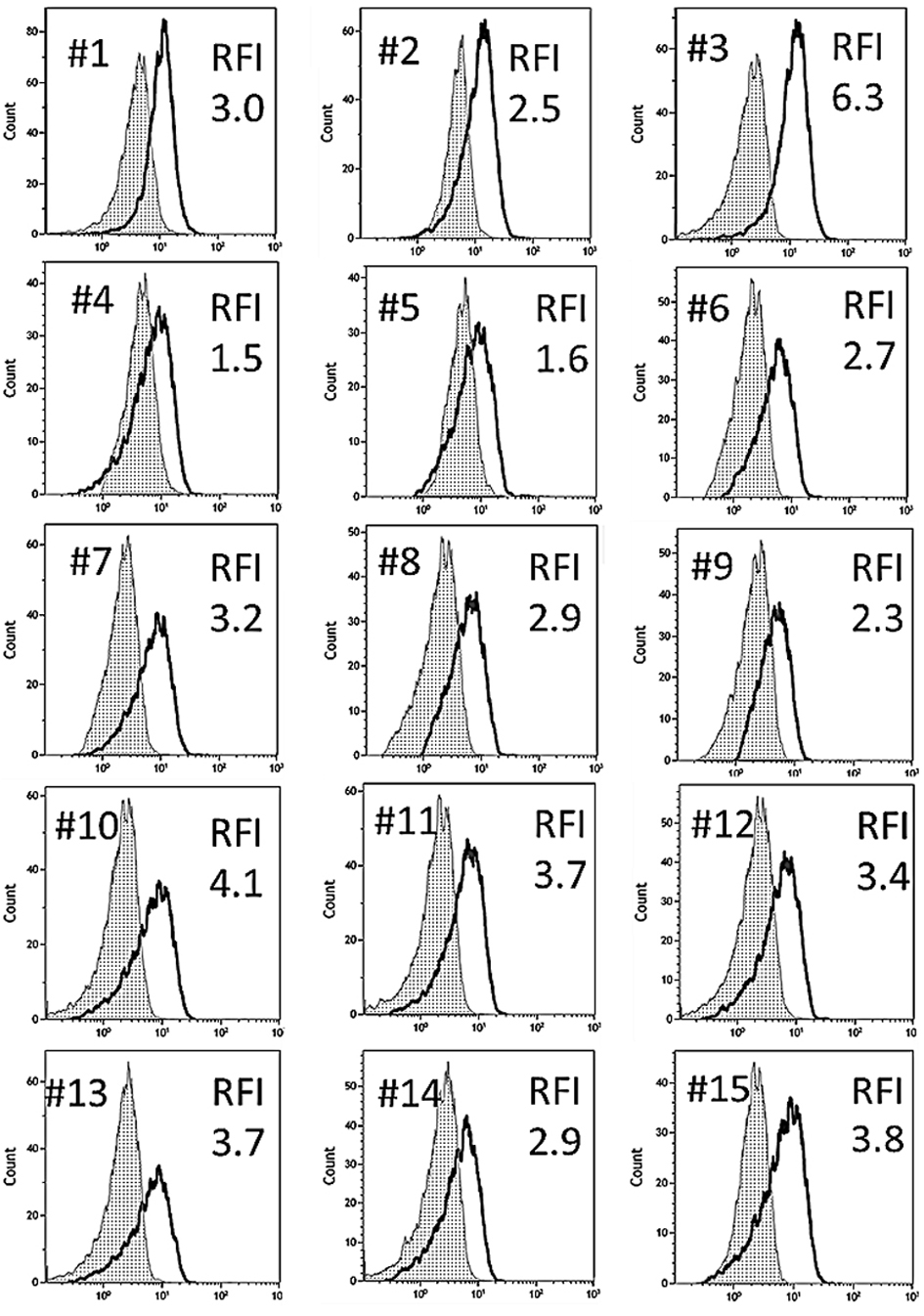

Supplement: Supplementary file 1 — Supplementary Figure S1. On‐bead flow cytometry for sEV in fraction #4 isolated from plasma of each melanoma patient included in this study (n = 15). sEV were immunocaptured on streptavidin beads using biotin‐labeled anti‐CD63 mAb as previously described (Sharma et al., 2018). Detection was performed using PE‐labeled anti‐CSPG4 mAb. Relative fluorescence intensity (RFI) values differ among patients but CSPG4+ sEV are present in total plasma‐derived vesicles of all 15 patients. [file JEV2-10-e12063-s001.jpg]

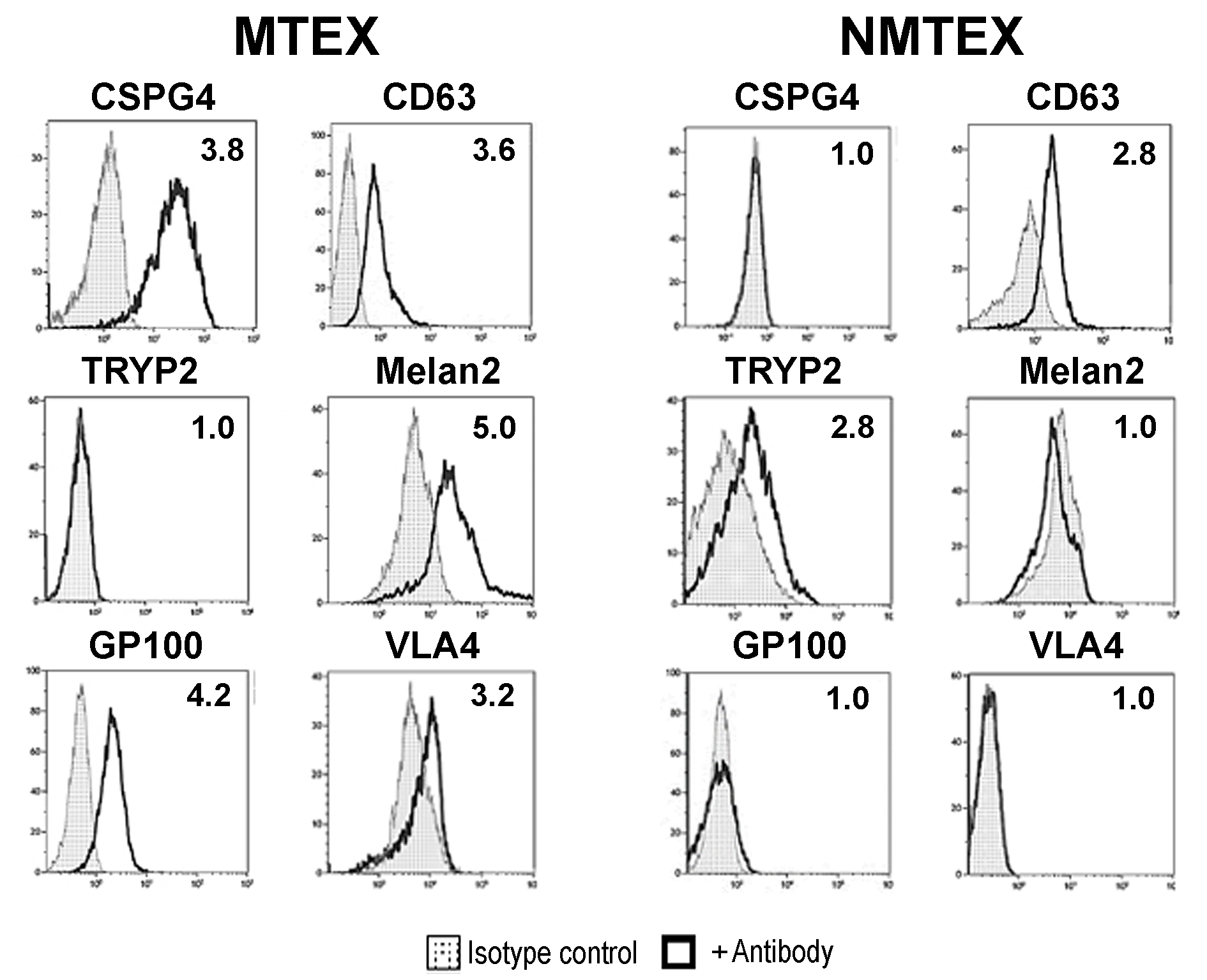

Supplement: Supplementary file 2 — Supplementary Figure S2. On‐bead flow cytometry for detection of melanoma‐associated antigens (MAAs) on the surface of MTEX or NMTEX. Relative fluorescent intensity (RFI) values for each antigen are marked. MTEX are enriched in MAA relative to NMTEX as previously reported in (Sharma et al., 2020). [file JEV2-10-e12063-s002.jpg]

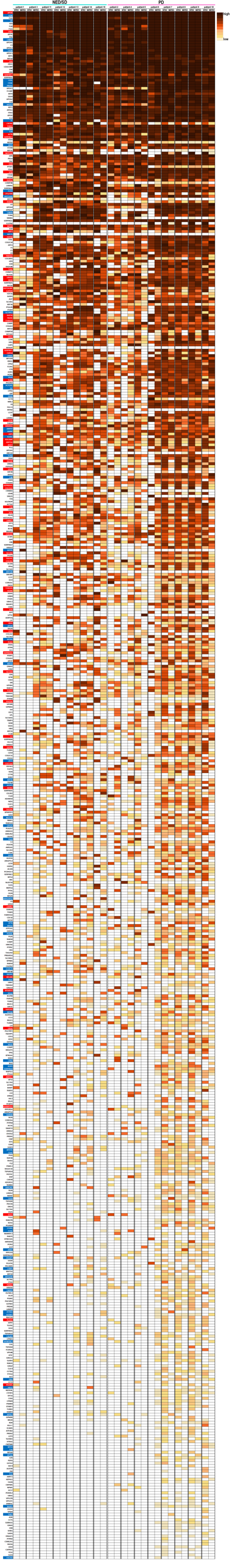

Supplement: Supplementary file 3 — Supplementary Figure S3. The heat map representing the abundance of 573 proteins detected in MTEX and NMTEX samples of 15 MM patients. NED/SD patients and PD are grouped. Names of MTEX‐upregulated and MTEX‐downregulated proteins are highlighted in red and navy blue, respectively. The abundance of proteins is colour‐coded according to deciles of all normalized signals. [file JEV2-10-e12063-s003.jpg]

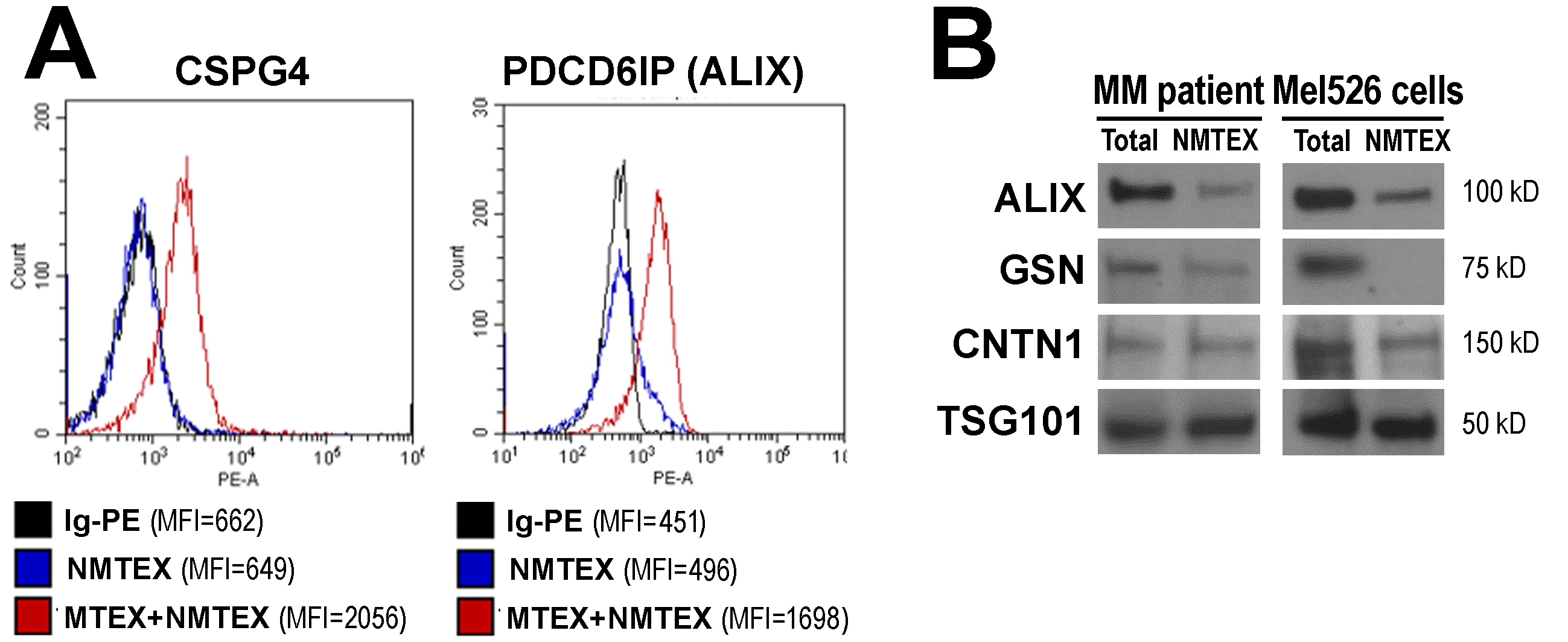

Supplement: Supplementary file 4 — Supplementary Figure S4. The detection of selected MTEX‐upregulated proteins in sEV from melanoma. Panel a – quantitation of CSPG4 and PDCDIP (ALIX) relative levels by the on‐latex bead flow cytometry in the mixture of MTEX and NMTEX as well as NMTEX alone isolated from plasma of a MM patient; marked is relative fluorescence intensity (RFI) including isotype control (Ig‐PE). Panel b – Western blot analysis of ALIX, gelsolin (GSN), contactin‐1 (CNTN1), and TSG101 in sEV isolated from plasma of a MM patient and supernatant of Mel526 melanoma cell line; represented is the mixture of MTEX and NMTEX (Total) as well as NMTEX alone. [file JEV2-10-e12063-s004.jpg]

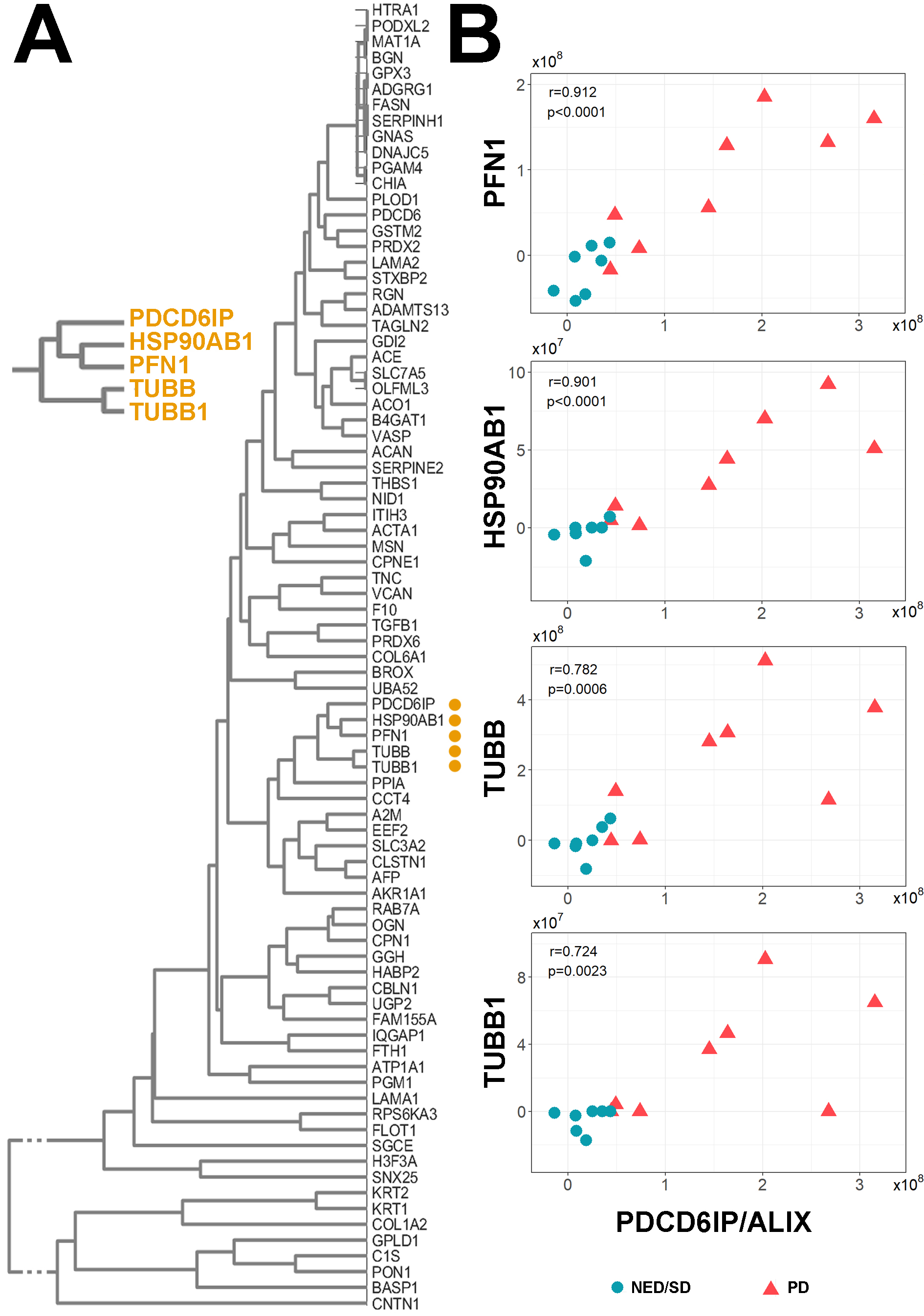

Supplement: Supplementary file 5 — Supplementary Figure S5. The correlation between MTEX proteins discriminating MM patients with NED/SD and PD. Panel a – the correlation tree of 83 MTEX proteins which differential (MTEX‐NMTEX) level was significantly different (p < 0.05) between NED/SD and PD; proteins which level was highly correlated with the level of PDCDI6P are marked with orange dots and presented in the insert. Panel b – the pairwise correlation between PDCDI6P and 4 proteins: PFN1, HSP90AB1, TUBB, and TUBB1). [file JEV2-10-e12063-s005.jpg]
